# Ni-alginate hydrogel beads for establishing breakthrough curves of lead ions removal from aqueous solutions

**DOI:** 10.1007/s11356-022-21305-8

**Published:** 2022-06-21

**Authors:** Nesreen M. Sami, A. A. Elsayed, M. M. S. Ali, Sayed S. Metwally

**Affiliations:** Egyptian Atomic Energy Authority, Hot Laboratories and Waste Management Center, Cairo, 13759 Egypt

**Keywords:** Alginate beads, Environmental protection, Fixed-bed column, Lead ions, Breakthrough modeling

## Abstract

The scientific impact of this work is the protection of the environment from hazardous pollutants using a column technique. Besides its higher stability at working pH and its time persisting, Ni-alginate has a higher ability to remove lead ions compared to the other prepared beads (Sr-alginate, Co-alginate, and Ca-alginate). Also, Ni-alginate possessed a higher removal percent, 93.3%, toward Pb^2+^ than the other ions, taking the sorption order of Pb^2+^ > Sr^2+^ > Co^2+^ > Cd^2+^ > Zn^2+^. Therefore, this study focused on using Ni-alginate as a selective sorbent for lead ions. Fixed-bed column was employed for the sorption process. The results for that efficiency are presented as breakthrough curves in view of the impact of various parameters; influent flow rate (1.5, 3.0, and 5.0 mL/min), lead concentration (100, 150, and 200 mg/L), and bed depth of sorbent (3.0, 5.0, and 7.0 cm). Breakthrough modeling including Thomas and Yan models was employed. The outcomes indicated that Thomas theory is more applicable. The overall outcomes indicated that Ni-alginate is recommended for selective removal of Pb^2+^ from waste solutions.

## Introduction

Radioactive and industrial liquid wastes comprise huge quantities of radionuclides and heavy metals like cadmium, mercury, zinc, and lead that have serious consequences on the environment if not treated accurately (Liang et al. 2019; Guo et al. 2018). The bioaccumulation of these pollutants may irrecoverably damage the biological machinery of creatures. Therefore, they should be treated to gain a safe environment.

Lead has several radioisotopes as ^210^Pb with a half-life of 22.3 years; it emits both α particles and γ radiation (Weng et al. 2020). Originally, ^210^Pb is produced by the decay chain of the naturally occurring ^238^U (Owen et al. 2021). Lead–210 is diffused in air, water, and soil and its accumulation may lead to carcinogenic dangers to people and unfriendly environmental impacts (Bonczyk, 2013). Lead is a considerably common heavy metal with high toxicity, and its ingestion may destroy the central nervous system, kidney, and digestive system (Ghaly et al. 2018). The fundamental job in the anticipation of contamination by lead is played by wastewater treatment before release into the environment. The dismissing of Pb^2+^ from wastewater is accordingly of specific significance. According to the instructions of WHO, the greatest admissible restriction of lead is 10 ppb, and zero lead centralization of water is favorable (Naga Babu et al. 2017). Additionally, lead is not biodegradable, and consequently, the issues are enhanced.

A portion of the attributes of adsorption-based methods is the utilization of normal, modest, non-poisonous, biocompatible, and biodegradable polymers like cellulose, chitosan, and alginate as possible adsorbents. One such polymer is sodium alginate which is a natural polysaccharide, generally presented in brown seaweed, made out of β-mannuronic and α-guluronic acid repeating monomers, gets cross-linked within the sight of di- or trivalent metal ions, and are employed as heavy metals adsorbent (Sneha et al. 2021). It is water dissolvable producing a jelly and interacts with divalent cations in an aqueous solution as Ca^2+^ in CaCl_2_ forming hard material named hydrogel (Metwally et al. 2020a). Hydrogels have intriguing highlights as simple to apply and ready to adsorb ions and forestall their loss; in this manner, these hydrogels have numerous utilizations particularly in water treatment (Han et al. 2021; Huan et al. 2021).

In the literature, different metal-alginate hydrogels were synthesized as sodium alginate (Xiaolin et al. 2021), calcium alginate (Soltani et al. 2020; Papageorgiou et al. 2009), and zinc alginate (Straccia et al. 2015; Abi Nassif et al. 2020). Nickel alginate composites are operative for dismissing of radionuclides from waste solutions (Oritani and Mimura, 2003). The high polar groups (–COOH and –OH) on alginate chains could go about as the coordination sites for anchoring targeted multivalent ions, particularly for Pb^2+^ with excellent physicochemical properties (large covalent index and small hydrated radius) via chemical and physical interactions, has been favored rather than other supporting materials presently (Shufeng et al. 2020). The divalent ions (Sr^2+^, Cu^2+^, Ni^2+^, Ba^2+^, Zn^2+^, Ca^2+^, etc.) link to two carboxyl groups on adjoining alginate molecules producing a three-dimensional network structure (Wang et al. 2020).

The alginate bead diameter and strength are controlled by Ca^2+^ solution concentration and time of reaction. Commonly, a long reaction time leads to a decrease in the bead diameter and forms more rigid beads resulting from further extensive alginate crosslinking and increasing the Ca^2^ concentration leads to constriction of the gel network (Metwally et al. 2020a).

Among all activities for the elimination of radionuclides and heavy metal ions from wastewater, solid bed extraction, precipitation, adsorption, and other techniques using different suitable materials in both batch and fixed-bed column. Batch sorption outcomes are valuable to provide data about the efficiency of sorbent and define numerous parameters of physicochemical procedures and the optimum conditions. However, the experimental outcomes attained from the batch cannot be applicable in a dynamic system where the contact time in the continuous system is permanently less than equilibrium time and this data is unqualified to give accurate information for scale-up objectives. Hence, the dynamic sorption system is more proper in detecting the maximum sorption capacity and sorbent employment in a real industrial-scale application.

In this work, Nickel-alginate was prepared and employed for the elimination of Pb^2+^ from the aqueous phase by a fixed-bed column. Different factors were examined like the impact of the flow rate of influent, impact of bed depth, and impact of Pb^2+^ concentrations and compared the data with predictions of Thomas and Yoon-Nelson models.

## Experimental

### Materials

All the materials utilized were of AR grade. Sodium alginate and chloride salts of lead, cobalt, strontium, and nickel were acquired from Sigma-Aldrich, Germany, and used without any treatment. The pH was adjusted by HCl and/or NH_4_OH which were purchased from Fluka. Water is used as deionized water.

### Alginate bead preparation

**Scheme 1:**
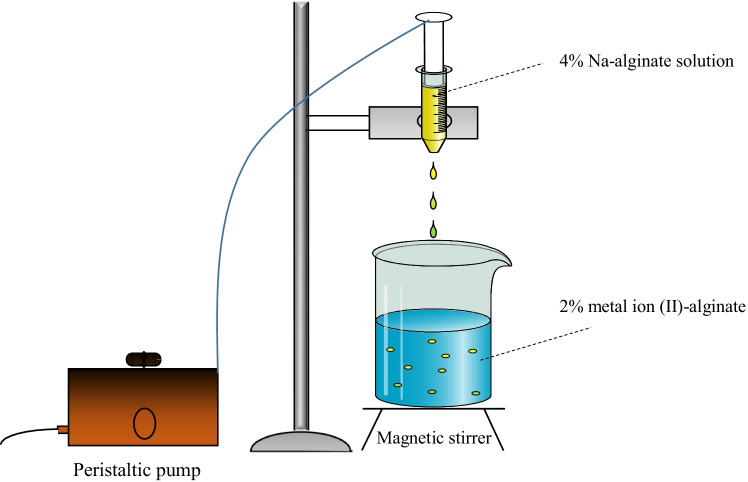
Preparation of metal (II)-alginate hydrogel beads

Different types of alginate hydrogel beads were produced by varieties of ions as crosslinkers, Sr, Co, Ca, and Ni ions to produce Sr-alginate, Co-alginate, Ca-alginate, and Ni-alginate, respectively. They were produced by a solution of 4% w/v of sodium alginate by dissolving 4 g of sodium alginate in 100 mL of deionized water with stirring for 24 h. The solution was left 12 h to degas, the alginate was added dropwise by a peristaltic pump into 200 mL of 2% (or 0.2 mol/L) aqueous metal ions (Sr^2+^, Co^2+^, Ca^2+^, and/or Ni^2+^) chloride solution at room temperature with gentle stirring by a magnetic stirrer for 30 min gelation period and attain equilibrium between metal cations (II) in solution and the ions sorbed on the beads, where the ions were bonded to the alginate beads. The diameter of the resultant beads was 1.5 ± 0.01 mm. The spherical beads were rinsed with distilled water several times and dried in the air. Scheme 1 displays the method of metal-alginate preparation.

### Column studies

Fixed-bed column sorption experiments were achieved in 1.0 cm of inner diameter glass column and 10.0 cm in length. The column was loaded with various weights (3.0, 5.0, and 7.0 cm) of Ni-alginate for altered bed depth. At the top end of the column, a piece of glass wool was placed to avoid the particles from floatation then the column was pretreated with deionized water in a downward flow direction to withdraw the trapped air between the beads at a certain pH (pH = 6.0 ± 0.1) and 25 °C till the pH value of the effluent becomes similar to the value of influent pH. The lead solution was delivered by a peristaltic pump at different flow rates (1.5, 3.0, and 5.0 mL/min) at room temperature. The ions solutions containing concentrations of 100, 150, and 200 mg/L of Pb^2+^ ions were fed to the column through a downflow. Samples were collected for analysis using Buck Scientific Atomic Absorption Spectrophotometer model 210 VGP, USA, air-acetylene flame. The flow continues until the effluent concentration (*C*_*eff*_) is almost equal to influent concentration (*C*_*o*_). The breakthrough curve displays the ions behavior sorbed through breakthrough and exhausted points which are set as *C*_*eff*_*/C*_*o*_ = 0.05 and *C*_*eff*_*/C*_*o*_ = 0.95, respectively. The column efficiency was assessed by the breakthrough curve of the fixed-bed structure. Commonly, the breakthrough curve can be plotted as the ratio of outlet concentration to input concentration, *C*_*eff*_*/C*_*o*_, as a function of the operational time *t*. The lead quantity sorbed, mg, was calculated from the following formula.

1 $$q_{tot} = \frac{QA}{{1000}} = \frac{Q}{1000}\int_{t = 0}^{{t = t_{tot} }} {C_{ads} dt = \frac{Q}{1000}\int_{0}^{{t = t_{tot} }} {(C_{o} - C_{eff} )dt} }$$

where *q*_*tot*_ is the total quantity sorbed of lead, mg, *Q* is the flow rate of influent, mL/min; *A* is the area under the breakthrough curve obtained by integration of the sorbed ion concentration (*C*_*ads*_ = difference between the influent and effluent concentrations, mg/L); and *t*_*tot*_ is the flow time, min. The lead ions breakthrough capacity, mg/g, was estimated from Eq. (2).

2 $${\text{Capacity}} \left( {{\text{mg}} /g} \right) = \frac{{q_{tot} }}{w}$$

where *w* is the weight of the bed, g. The total amount of ion fed to the column, m_tot_, (mg) and the column performance (total ion removal, %, by the column) are computed from the following equations.

3 $$m_{tot} = C_{o} \frac{{V_{eff} }}{1000}$$

where *V*_*eff*_ is the total volume of the effluent, mL.

4s $${\text{Total}} ion removal(\% ) = \frac{{q_{tot} }}{{m_{tot} }}{\text{x}} 100$$

## Results and discussion

**Fig. 1:**
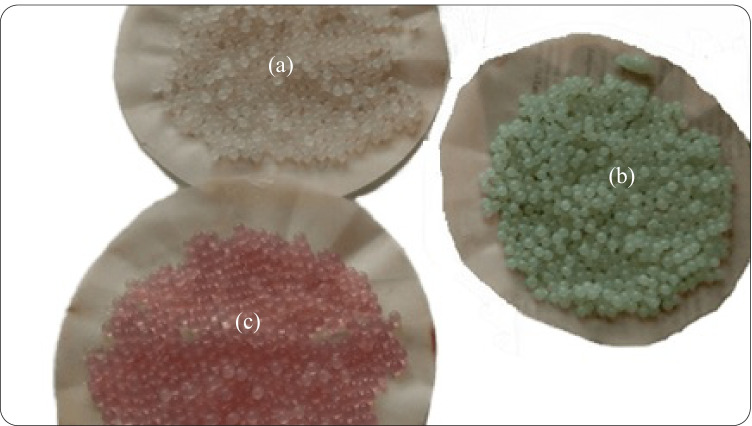
The spherical shape of the prepared beads **a** Sr-alginate, **b** Ni-alginate, and **c** Co-alginate

The prepared alginate beads (Sr-alginate, Co-alginate, Ca-alginate, and Ni-alginate) were tested for sorption of different ions from aqueous solutions; the results were compared with each other as reported in Table 1. Besides its higher stability at working pH and its time persisting, Ni-alginate has a higher ability to remove lead ions compared to the other prepared beads. Also, Ni-alginate possessed a higher removal percent, 93.3%, toward Pb^2+^ than the other ions, taking the sorption order of Pb^2+^  > Sr^2+^  > Co^2+^  > Cd^2+^  > Zn^2+^. Therefore, this study focused on using Ni-alginate as a selective sorbent for lead ions. Figure 1 also displays the sphere beads shape of the different beads.

**Table 1:** Comparison of removal percent of different ions by different alginate beads

Elements	Removal percent, %			
	Sr-alginate	Co-alginate	Ni-alginate	Ca-alginate
Cs	35.1	30	28.7	33
Pb	**78.2**	**85.7**	**93.3**	**89**
Zn	10.1	9.4	7.6	20
Sr	16.4	55.6	62.5	65
Cd	12.7	12	11.8	15
Co	35.2	22.8	33.5	40

The bold font means the ion under study in this article

### Characterization of Ni-alginate

**Fig. 2:**
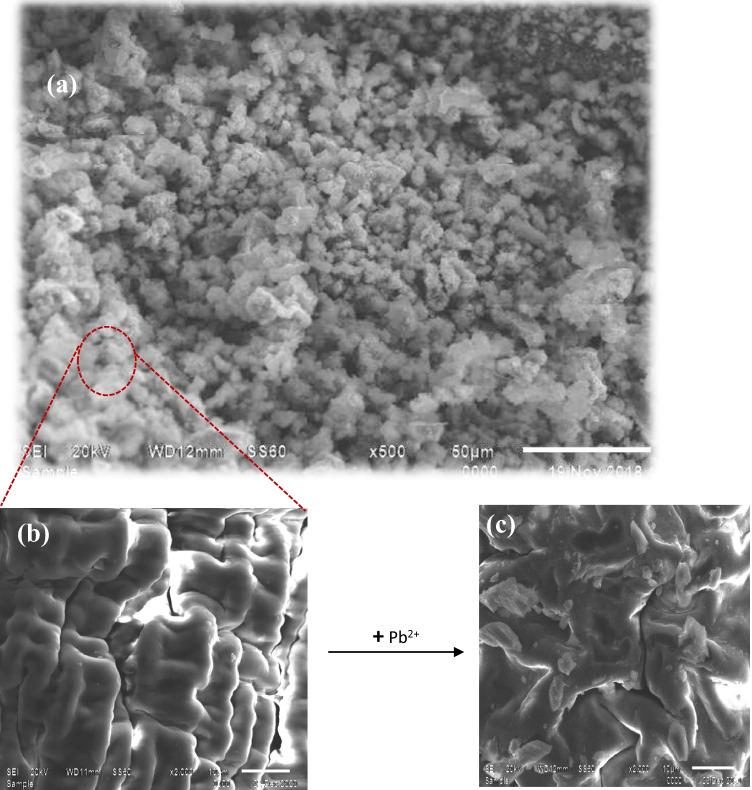
SEM images of **a** Ni-alginate (500 ×), **b** Ni-alginate (2000 ×), and **c** Ni-alginate-Pb

As mentioned above, Ni-alginate was selected for further studies; therefore, the Ni-alginate morphology was studied using scanning electron microscope as displayed in Fig. 2. The spherical shape of Ni-alginate is illustrated in Fig. 2a with an average size of ~ 12 µm. This figure displays that Ni-alginate has a rugged surface, is highly dense with some wrinkles, and is porous. By increasing the magnification power, the layers with cavities of Ni-alginate were observed (Fig. 2b). After the sorption of Pb^2+^, the cavities were decreased due to the sorption process (Fig. 2c).

**Fig. 3:**
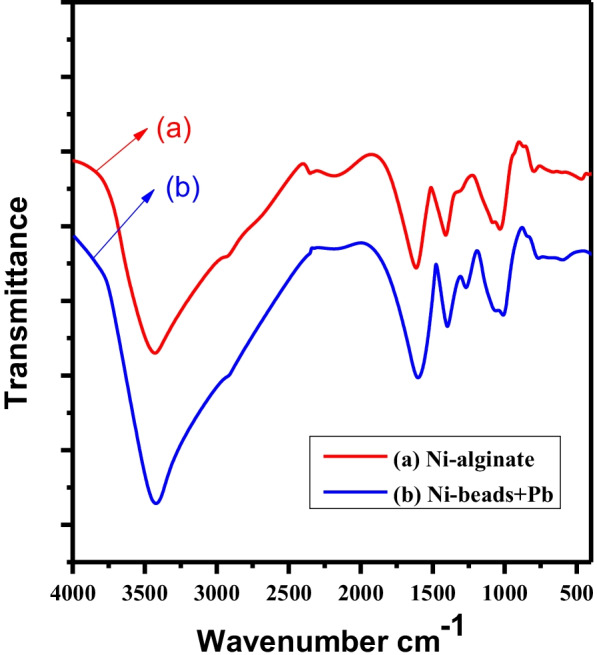
FT-IR spectra of **a** Ni-alginate and **b** Ni-alginate-Pb

The infra-red bead spectrum is displayed in Fig. 3. The broadband centered at 3000–3600 cm^−1^ is due mainly to the stretching vibration of the –OH group. The band around 1024 cm^−1^ is ascribed to C–O–C stretching of alginate beads. The bands at 1586 and 1405 cm^−1^ are assigned to the asymmetric and symmetric C = O stretches of the carboxylate group, respectively (Daradmare et al. 2020). The bands indicate the existence of polysaccharides in the alginate (Sujana et al. 2013). The difference in the spectral peaks before and after lead sorption is noticed in the appearance of a small peak at 1385 cm^−1^ in the spectrum after sorption due to O–Pb group (Naga Babu et al. 2017). The appearance of a new peak after Pb^2+^ sorption demonstrates that lead ions were replacing the nickel ions, forming the Pb-alginate complex (Jing et al. 2019). Also, the peak strength increased markedly after sorption of Pb^2+^; this is compatible with the results obtained in the literature (Wu et al. 2019).

### Mechanism of sorption process

**Scheme 2:**
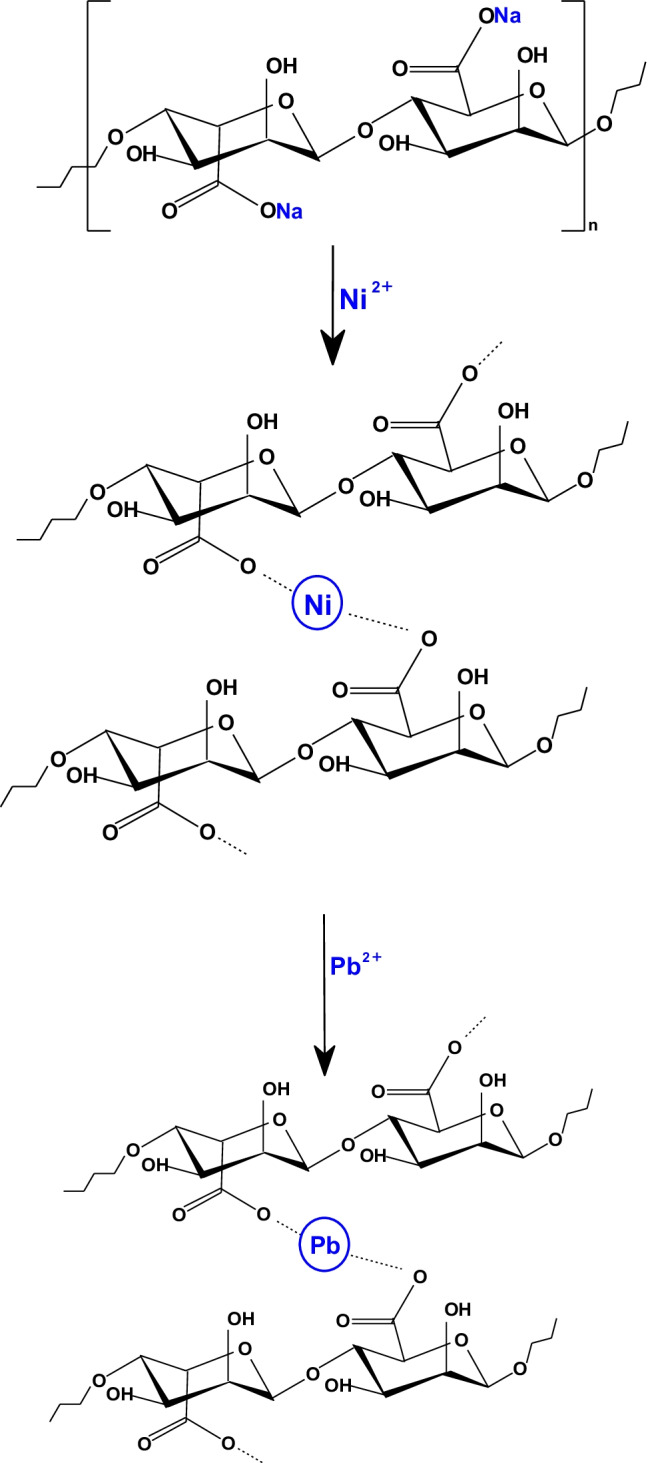
Proposed mechanism for sorption of lead ions onto Ni-alginate hydrogel beads

Scheme 2 displays the possible mechanism for the preparation of Ni-alginate and the sorption of Pb^2+^. The high polar carboxylate groups in alginate molecule act as coordination sites for Ni^2+^; therefore, Ni^2+^ links to two carboxylate groups on adjoining alginate molecules (Wang et al. 2020). The suggested sorption mechanism indicates that the sorption occurs by ion exchange process, by replacing the nickel ions with the lead ions. This was confirmed by analyzing the filtrate after sorption; the outcomes revealed that it contains Ni^2+^ ions; this confirms that the sorption process is controlled by ion exchange as displayed by Scheme 2.

### Column studies

Breakthrough curves can be gained under different conditions via plotting C_eff_/C_0_ and time. These conditions include the impact of input flow rate, bed depth of Ni-alginate, and lead ions concentration as follows.

#### Effect of influent flow rate

**Fig. 4:**
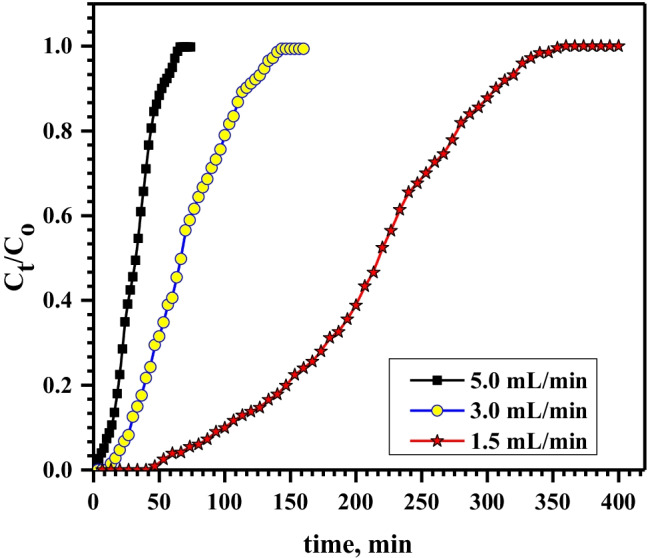
Effect of flow rate on sorption of 100 mg/L Pb.^2+^ onto 3.0 cm bed depth of Ni-alginate

The dynamic behavior and column performance for Pb^2+^sorption were investigated by varying the input flow rate from 1.5 to 5.0 mL/min at a lead initial concentration of 100 mg/L and Ni-alginate bed depth of 3.0 cm as displayed in Fig. 4. It illustrates that the time needed to achieve the breakthrough point diminishes with expanding the flow rate. In addition, at 5 mL/min flow rate, the breakthrough curve was steeper as compared to 1.5 and 3.0 mL/min. At the lowest flow rate (1.5 mL/min), the breakthrough time was gained after 70 min while at the highest flow rate (5.0 mL/min), the breakthrough time was 8 min. The sorbent capacity is maximum when the flow rate is minimum (Table 2). This is attributed to the longest contact time between Ni-alginate particles and Pb^2+^ solution which simplified the mass transfer rate.

**Table 2:** Effect of flow rate, bed depth, and ions concentrations on the capacity and column performance for sorption of Pb.^2+^ by Ni-alginate

Factor		q_total_, mg	m_tot_, mg	Column performance, %	Capacity, mg/g
Flow rate, mL/min	1.5	31.5	54	58.3	15.7
	3.0	24.0	43	55.8	12.0
	5.0	17.5	33	53.0	8.8
Bed depth, cm	3.0	31.5	54	58.3	15.7
	5.0	49.5	65	76.1	15.9
	7.0	66	76	86.8	16.1
Conc., mg/L	100	31.5	54	58.3	15.7
	150	36.5	61	59.8	18.3
	200	45.6	74	61.6	22.8

#### Effect of bed depth

**Fig. 5:**
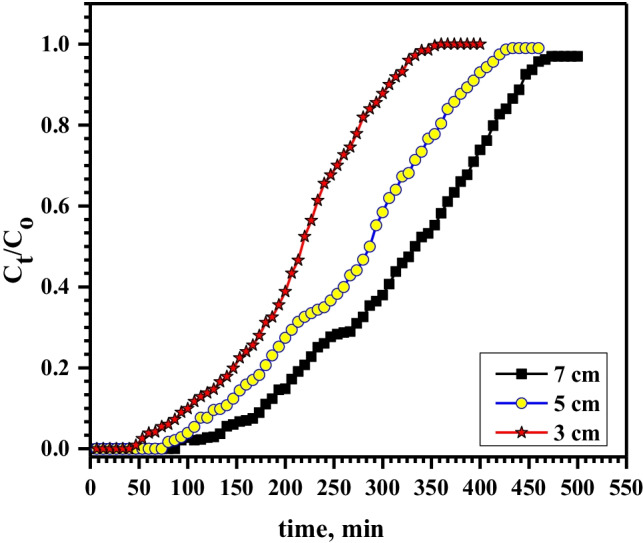
Effect of bed depth on sorption of 100 mg/L Pb.^2+^ onto Ni-alginate at flow rate of 1.5 mL/min

In column sorption investigations, the breakthrough point depends on the quantity of Ni-alginate beads and consequently, their bed height in the column. It is clear that the breakthrough time increments with expanding the bed depth (Fig. 5). The breakthrough point, 5%, increased from 70 to 135 min with increasing the bed depth from 3.0 to 7.0 cm; correspondingly, the exhausted point, 95%, increased from 320 to 460 min with increasing the bed height from 3.0 to 7.0 cm. This is ascribed to the increase of active sites and the sorbent surface area (Metwally et al. 2020b). Accessible active sites for Pb^2+^ binding were also less so that column exhaust time arrived earlier and the saturation rate was additionally faster at 3.0 cm bed depth. Whereas at higher bed height, the mass transfer zone is widened, the time of contact between Pb^2+^ and the Ni-alginate increased, and the number of accessible active positions for the Pb^2+^ uptake also increased, which caused the longer exhaustion and breakthrough times and therefore, the capacity and column performance increased (Table 2).

### *Effect of Pb*.^*2*+^*ions concentration*

**Fig. 6:**
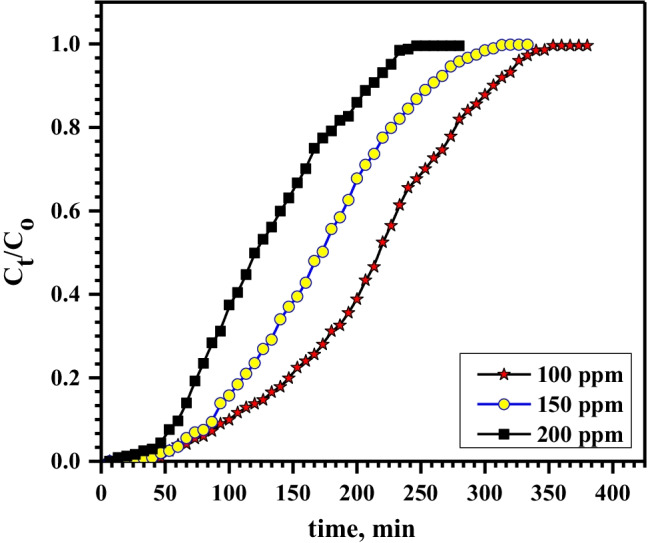
Effect of Pb.^2+^ concentration on its sorption onto Ni-alginate at flow rate of 1.5 mL/min and 3.0 cm bed depth

The influence of Pb^2+^ concentration on its sorption by Ni-alginate was tested by varying the lead initial concentration from 100 to 200 mg/L at 3.0 cm bed depth and influent flow rate of 1.5 mL/min. The breakthrough curve was gained by plotting C_eff_/C_o_ against *t* (Fig. 6). At the highest lead concentration, the breakthrough curve was sharper and the time required for breakthrough and exhaustion decreased while the column performance and capacity increment with increasing the lead concentration, as reported in Table 2. This behavior is due to that the active sites are quickly saturated at the highest concentrations. Whereas at the lowest concentration, the breakthrough and exhaustion time gained slowly; this is due to a decreased mass transfer coefficient. The results demonstrated that the breakthrough time decreased from 70 to 50 min with increasing the influent concentration from 100 to 200 mg/L, while the capacity increased from 15.7 to 22.8 mg/g.

### Modeling of breakthrough curves

Mathematical models display an essential role in computing the sorption capacity of metal ions. The contaminated solution passed through a fixed-bed of Ni-alginate at defined bed depth, flow rate, and ions concentration. Attempts were executed, established on experimental outcomes, to promote a technique that defines the removal of the ions by the fixed-bed of Ni-alginate. Numerous models are applied to characterize the breakthrough curves in the fixed-bed column such as Thomas and Yan models; they suggested estimation of the breakthrough curve stated from a generalized logistic function that included, as follows.

#### Thomas model

**Fig. 7:**
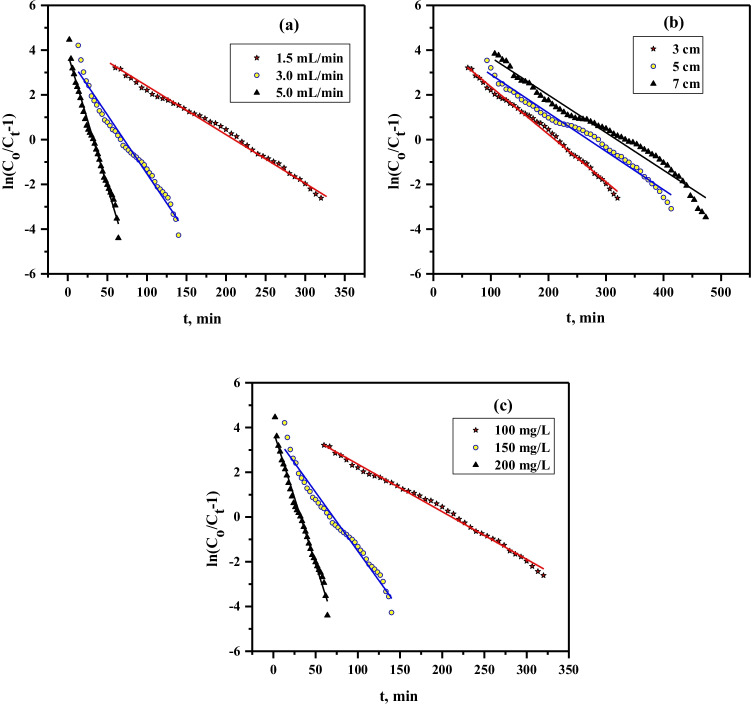
Plot of Thomas model for Pb.^2+^ sorption at different **a** flow rates, **b** bed depths, and **c** ions concentration by Ni-alginate

Thomas model is employed to expect the capacity and rate of adsorption by the continues-flow column. This model suggested that the sorption is limited by mass transfer at the interface (Yasar Recepoglu et al. 2018). The mathematical formula of Thomas theory is formulated by Eq. (5) (Thomas, 1944) and fitted with the practical outputs gained from the continues-flow column experiments.

5 $$\ln \left( {\frac{{C_{o} }}{{C_{t} }} - 1} \right) = \frac{{q_{Th} k_{Th} m}}{Q} - k_{Th} C_{o} t$$

where *k*_*Th*_ and *q*_*Th*_ are Thomas rate constant, mL min^−1^ mg^−1^, and the capacity of sorbed lead ions, mg/g, respectively. The plot of *ln((C*_*o,*_*/C*_*t*_*)-1)* vs *t* gives straight lines (Fig. 7); Thomas parameters are computed from the slope and intercept and stated in Table 3. At the highest flow rate (5 mL/min), *k*_*Th*_ has the greatest value; this is resulting from the high mass transfer rate (Rokhsare et al. 2018). While the *q*_*Th*_ has the lowest value at the highest flow rate; this is due to inadequate time for sorption and diffusion of Pb^2+^. With regard to the bed depth, the outcomes demonstrated that the increase of bed depth reduces the *k*_*Th*_ due to long contact time at higher bed depth. Whereas the *q*_*Th*_ enlarged with increasing the bed depth this is resulting from increasing the sorption sites. Regarding the inlet concentration, both the k_Th_ and q_Th_ values enlarged with rising the lead ions concentration; this is resulting from the increase of lead ions quantity exposed to the sorption. The values of q_Th_ are reasonable with q_experimental_ and all values of the correlation coefficient are R^2^ > 0.96; this indicates that the Thomas model is reasonable to predict the Pb^2+^ sorption by Ni-alginate in the continues-flow column. These results are in agreement with those reported in the literature (Ahmad and Hameed, 2010; Hui et al. 2017; Yuanyao et al. 2018).

**Table 3:** Thomas model parameters for Pb.^2+^ sorption by Ni-alginate at different bed depths, flow rates, and ions concentrations

Parameters	Flow rate, mL/min			Bed depth, cm			Ions concentration, mg/L		
	1.5	3.0	5.0	3.0	5.0	7.0	100	150	200
*k*_Th_, mL/mg/min	0.23	0.53	1.19	0.23	0.17	0.16	0.23	0.35	0.59
*q*_Th_ (Calculated), mg/g	15.75	10.66	8.08	15.75	16.15	17.65	15.75	17.94	19.84
*q* (Experimental), mg/g	15.70	12.01	8.80	15.70	15.9	16.10	15.70	18.30	22.80
R^2^	0.98	0.97	0.99	0.98	0.98	0.97	0.98	0.97	0.96

#### Yan model

**Fig. 8:**
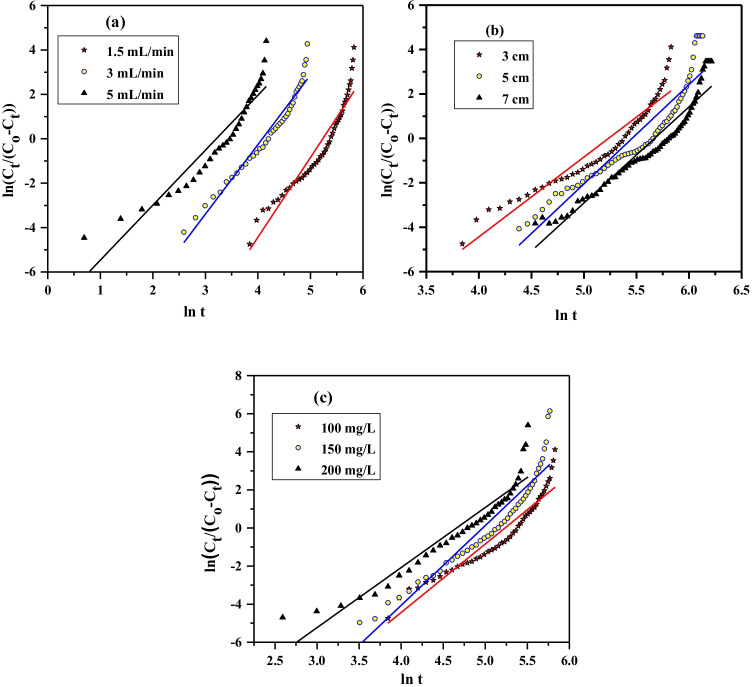
Plot of Yan model for Pb.^2+^ sorption at different **a** flow rates, **b** bed depths, and **c** ions concentration by Ni-alginate

Yan et al. (2001) modified Thomas theory to diminish the error resulting from using the Thomas equation, particularly at very short and very long operation times (Munmun et al. 2018, Espina de Franco. 2017); it is stated as,

6 $$\ln \left( {\frac{{C_{t} }}{{C_{o} - C_{t} }}} \right) = \frac{{k_{Y} C_{o} }}{Q}\ln \left( {\frac{{Q^{2} }}{{k_{Y} q_{Y} m}}} \right) + \frac{{k_{Y} C_{o} }}{Q}\ln t$$

where q_y_, mg/g, and K_y_, L mg min^−1^ are the adsorption capacity and Yan rate constant, respectively. The values of q_y_ and K_y_ can be computed from the intercept and slope of the plot of *ln(C*_*t*_*/(C*_*o*_*–C*_*t*_*))* versus *ln t* (Fig. 8). Table 4 illustrated that the *q*_*Y*_ values decreased from 5.36 to 2.44 mg/g with increasing the influent flow rate from 1.5 to 5 mL/min while the k_*Y*_ value increased; this is resulting from the reasons mentioned above. For altered bed depths, the *q*_*Y*_ value increased with the increase of bed depth; this is in agreement with the data gained in the literature (Yuanyao et al. 2018). The *q*_*Y*_ values increased from 5.36 to 10.58 mg/g with increasing the lead ions concentration from 100 to 200 mg/L. The values of correlation coefficient are R^2^ > 0.88 and the *q*_*Y*_ values are not reasonable with *q* (experimental) values. Comparing the data gained from Thomas with that of Yan model declared that the Thomas model is reasonably well than Yan to describe the Pb^2+^ sorption by Ni-alginate with a lower difference between the calculated and experimental values of the amount sorbed of lead ions.

**Table 4:** Yan model parameters for Pb.^2+^ sorption by Ni-alginate at different bed depths, flow rates, and ions concentration

Parameters	Flow rate, mL/min			Bed depth, cm			Ions concentration, mg/L		
	1.5	3	5	3	5	7	100	150	200
*k*_Y_, mL/mg/min	51.19	93.90	124.0	51.19	67.05	64.95	51	41.8	23.63
*q*_Y_ (Calculated), mg/g	5.36	2.84	2.44	5.36	6.52	7.44	5.36	8.52	10.58
*q* (Experimental), mg/g	15.70	12.01	8.80	15.70	15.9	16.10	15.70	18.30	22.80
R^2^	0.90	0.95	0.88	0.90	0.88	0.92	0.90	0.90	0.89

## Conclusion

Different alginate beads were successfully synthesized such as Ni-alginate, Sr-alginate, Co-alginate, and Ca-alginate. Ni-alginate beads have a higher ability to adsorb lead ions compared to the other beads. Ni-alginate beads were employed for sorption of different metal ions taking the adsorption order of Pb^2+^  > Sr^2+^  > Co^2+^  > Cd^2+^  > Zn^2+^. Ni-alginate is a selective sorbent for lead ions. The breakthrough capacity increases with increasing both the bed depth and ions concentration and decrease with increasing the flow rate. Breakthrough modeling including Thomas and Yan models was employed. Thomas model is more applicable with a high correlation coefficient (R^2^ > 0.96). Ni-alginate hydrogel is recommended for the selective removal of lead ions from waste solutions.

## Data Availability

All data generated or analyzed during this study are included in this published article.
